# 3D skeletal muscle model recapitulating the myostatin knockout phenotypic and mitochondrial metabolic features

**DOI:** 10.14814/phy2.70947

**Published:** 2026-06-16

**Authors:** Barbara Vernus, Elodie Jublanc, Béatrice Chabi, Laurence Pessemesse, Zacharie Cheng‐Boivin, Benoit J. Gentil, Anne Bonnieu, Christelle Koechlin‐Ramonatxo, Bénédicte Goustard

**Affiliations:** ^1^ DMEM Univ Montpellier, INRAE Montpellier France; ^2^ Department of Kinesiology and Physical Education McGill University Montreal Quebec Canada; ^3^ Department of Neurology and Neurosurgery and Montreal Neurological Institute McGill University Montreal Quebec Canada; ^4^ Sylvan Adams Sport Science Institute McGill University Montreal Quebec Canada

**Keywords:** 3D skeletal muscle, mitochondrial respiration, murine myoblasts/myotubes, myogenic differentiation, myostatin deficiency

## Abstract

3D cell culture, using a variety of bioengineering techniques, enables muscle cells to be cultured in more structural and functional biomimetic conditions than 2D cell culture. Here, we tested the ability of an engineered 3D skeletal muscle model to recapitulate *in vivo* metabolic muscle response. First, C2C12 myoblasts in 3D cultures showed improved myogenesis, attested by increased differentiation time, myotube formation, and gene expression of differentiated muscle markers. At the functional level, the 3D muscle culture displayed contractile properties and proper mitochondrial respiration. Second, to highlight the interest of such system we used primary myoblasts derived from myostatin knockout (*Mstn* ^−/−^) mice. When compared to control wild‐types 3D myotubes, 3D myotubes made from *Mstn* ^*−/−*^ myoblasts exhibit a hypertrophic phenotype associated with a decrease mitochondrial oxygen consumption, consistent with the skeletal muscle characteristics of *Mstn* ^*−/−*^ mice. Our findings show that 3D primary myotubes retain their *in vivo* phenotype in culture. This provides a useful framework for studying the underlying mechanisms of a various genetic muscle diseases, as well as for screening therapeutic drugs.

## Graphical abstract

3D muscle culture reproduces the metabolic characteristics associated with the myostatin knockout phenotype.

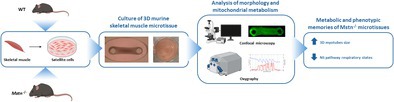

## 1 INTRODUCTION

Skeletal muscle is the largest tissue mass in the body and plays a central role in posture, voluntary movements, and the protection of soft tissues and body openings. Beyond these mechanical functions, it also contributes to key metabolic and homeostatic processes.

One of the growth factors regulating muscle growth and metabolism is myostatin. Myostatin, a member of the TGF‐beta family, is expressed in skeletal muscle and functions to limit growth of muscle. Deletion of the myostatin gene results in excessive growth of skeletal muscle that can reach up to 200% (Lee, 2004; McPherron et al., 1997). Furthermore, a number of studies indicated a reduced muscle performance with lack of myostatin, attested by increased fatigability and aerobic metabolic deficit (Amthor et al., 2007; Rehfeldt et al., 2005; Wegner et al., 2000). Our previous studies showed that in vivo myostatin knockout (*Mstn* ^*−/−*^) muscles exhibit mitochondrial dysfunction, early fatigue, and poor recovery, both in slow (soleus) and fast (Extensor digitorum longus, EDL) muscles (Ploquin et al., 2012). At the metabolic level, *Mstn* ^*−/−*^leads to reduced mitochondrial coupling efficiency due to increased oxygen consumption at basal level, without changes in fiber type composition. Collectively, these findings highlight that myostatin not only controls muscle size but also governs its oxidative and mitochondrial metabolism, linking muscle growth to energy efficiency.

Animal models have been instrumental in elucidating the molecular mechanisms underlying these processes. However, in vivo models present methodological and ethical limitations, making in vitro 3D muscle models particularly relevant for studying these processes in a controlled environment.

Among the available models, the murine myoblast cell line C2C12 is widely used in the skeletal muscle field (Burattini et al., 2004; Ikeda et al., 2017; McMahon et al., 1994). When exposed to low‐serum differentiation media, these cells fuse into multinucleated myotubes that express contractile proteins and, after prolonged culture, may even display spontaneous contractions. These properties make C2C12 cells a valuable tool for investigating muscle development at the molecular level. However, they still fall short of faithfully reproducing in vivo muscle fibers, contractile proteins often remain disorganized, aligned sarcomeres are rarely formed, and several signaling pathways are not representative of mature skeletal muscle (Deshmukh et al., 2015). Consequently, there is a pressing need to establish culture systems that promote further maturation of myotubes to better model in vivo skeletal muscle biology. To date, the 3D muscle model emerges as a promising intermediate approach that better mimics the structure and function of skeletal muscle tissue (Kasper et al., 2017; Khodabukus et al., 2007).

Compared to 2D cultures, human skeletal muscle 3D cultures exhibit enhanced mitochondrial maturation, improved extracellular matrix remodeling and differences in muscle fiber phenotype transitioning from slow‐ to fast‐twitch characteristics (Tollitt et al., 2025). Therefore, 3D engineered muscle tissue has great potential for reproducing the complexity of muscle physiology. Beyond structural and functional features, the next challenge is to develop models capable of faithfully recapitulating metabolic processes in muscle. In this context, one major limitation of current 3D skeletal muscle models is the lack of physiologically relevant mitochondrial function. Indeed, faithful in vitro mitochondrial respiration would provide a valuable tool to investigate the mechanisms underlying a broad range of diseases and to accelerate drug discovery.

The objective of this study was to establish a 3D skeletal muscle model using C2C12 myoblasts. To validate this model, we evaluated the morphological and histological characteristics of the resulting microtissue and analyzed the expression of myogenic markers during differentiation. We subsequently extended this approach to primary murine cultures derived from *Mstn* ^*−/−*^ mice, which display a specific muscle phenotype, to evaluate mitochondrial respiratory function.

## 2 MATERIALS AND METHODS

### 2.1 Materials

All chemical reagents were supplied by Sigma‐Aldrich (Saint Louis, MO, USA), unless otherwise specified: DMEM (D5796), pronase (P5147), fibrinogen (F8630), basic fibroblast growth factor (β‐FGF) (F0291), Ham's F‐10 (N6908), penicillin/streptomycin (P4458), amphotericin (A2942), pluronic® F‐127 (P2443), thrombin (T4393), 6‐aminocaproic acid (A2504), protease inhibitor cocktail (P8340), primary antibody α‐actinin sarcomeric (A7811), troponin T (T6277), fast myosin heavy chain (MHC) (M4276), hoechst (94403), digitonin (D141), pyruvate (P2256), malate (M0875), adenosine 5′‐diphosphate (ADP) (A5285), cytochrome c (C7752), glutamate (G1626), succinate (S2378), carbonylcyanide‐chlorophenylhydrazone (CCCP) (C2759), rotenone (R8875) and antimycin a (A8674).

For cell culture, fetal calf serum (FCS) was purchased from Dominique Dutscher (500105N1N, Bernolsheim, France), horse serum (HS) from PAN Biotech GmbH (*Aidenbach*, Germany), Matrigel® (354234) and 12‐well microplate‐Falcon (351143, Corning Life Sciences, Tewksbury MA, USA), and Geltrex (A14133‐02, LDEV‐Free hESC) from ThermoFisher Scientific‐Gibco (Waltham, MA, USA). For surface cellware, the OMEGA^MP^ device was provided by Enuvio (Montreal, Canada).

For immunofluorescence labelling, paraformaldehyde was procured from Electron Microscopy Sciences (15714, Hatfield, PA, USA), FluoProbes® 488 Goat Anti‐Mouse IgG (H + L) antibody from Interchim (FP‐SA4000, Montluçon, France).

For tissue Clearing and microscopy, RapiClear® reagent (IS216) and iSpacer microchamber were supplied by SUNJin Lab (Taiwan, ROC).

For gene expression studies (RT‐qPCR), the following reagents were obtained from Invitrogen: TRIzol reagent from Invitrogen (15596026, Carlsbad, CA), high‐capacity cDNA Reverse Transcription Kit from Applied Biosystems (4368813, Waltham, MA, USA), and Mastermix SensiFast SYBR Hi‐ROX kit from Meridian Bioscience (BIO‐92020, Tennessee, TN, USA).

For protein isolation and Western blot analysis, the BioRad DC kit was purchased from Biorad (500.0006, Hercules, CA, USA), embryonic MHC and sarcomeric MHC antibodies from DSHB (F1.652 and MF20, Iowa, IA, USA), peroxidase‐conjugated secondary antibody from Cell signaling (7076, Danvers, MA, USA), β‐actin antibody (sc‐81178) and ECL kit from Santa Cruz biotechnology (sc‐2048, Dallas, TX, USA).

### 2.2 C2C12 cell culture

The murine skeletal muscle cell line C2C12 was obtained from the American Type Culture Collection (ATCC CRL1772) (Manassas, VA, USA). Cells were grown in DMEM containing 10% FCS (v/v), 10 U/mL penicillin and 10 μg/mL streptomycin (growth medium (GM)) at 37°C in a humidified atmosphere of 5% CO_2_–95% air. Cells were kept in GM until confluence, then trypsinised and counted to generate muscle microtissues.

### 2.3 Primary mouse muscle cell culture

*Mstn* ^*−/−*^ male mice, harboring a constitutive deletion of the third *Mstn* exon, have been described previously (Grobet et al., 2003) and were generously provided by L. Grobet (Faculty of Veterinary Medicine, University of Liège, Belgium). *Mstn* ^*−/−*^ mice were generated in the C57BL/6J genetic background. Animals were maintained on a 12‐h/12‐h light/dark cycle and were allowed free access to food (A04, SAFE Diets, Augy, France) and water, and genotyped as previously described. Primary cultures of satellite cells were prepared from 4‐ and 6‐ week‐old male mice in accordance with institutional and national guidelines. Two wild‐type mice and one *Mstn* ^*−/−*^ mouse were used for each independent experiment, enabling the formation of five 3D skeletal muscle microtissues. Satellite cells were isolated from the whole muscles of the hindlimbs and culture protocol was performed as previously described (Rodriguez et al., 2014). Briefly, murine myoblasts were isolated from muscle after enzymatic digestion by pronase (Descamps et al., 2008; Levin et al., 2001). Cells were plated at a density of 2 × 10^4^ cells/cm^2^ on Matrigel®‐coated Petri dishes, in growth medium: 80% Ham's F‐10 supplemented with 20% HS (v/v), 10 U/mL penicillin, 10 μg/mL streptomycin and 0.5 μg/mL amphotericin. They were maintained at 37°C in a water‐saturated atmosphere containing 5% CO_2_‐95% air. After 2 days, cells were washed with Ham's F10 and placed in complete medium supplemented with 5 ng/mL basic fibroblast growth factor (β − FGF). Satellites cells were then grown 2 days more days and trypsinised (passage 1) and counted to generate muscle microtissues.

### 2.4 Culture of 3D skeletal muscle microtissues

An OMEGA^MP^ device was placed into a well of the included 12‐well microplate. The chambers of the device were coated with 5% Pluronic® F‐127 solution in phosphate buffer (PBS) (w/v) at 4°C for at least 12 h.

Satellites cells were suspended in a hydrogel mixture (Geltrex/fibrinogen [4 mg/mL]/GM, 1/2/2, v/v/v) at 25 × 10^6^ cells/ml at 4C on ice. Thrombin was added at 0.2 unit per mg of fibrinogen prior to seeding the cell/hydrogel mixture in the two chambers of the Enuvio device. Promptly, 40 μL of the hydrogel mixture were seeded into each chamber containing the micropillars.

The cell/hydrogel mixture was then incubated for 30 min at 37°C to expedite thrombin‐mediated fibrin polymerization to form a microtissue. C2C12 growth medium, containing 1.5 mg/mL 6‐aminocaproic acid, an antifibrinolytic agent, was then added to the well to completely submerge the device in its entirety. The next day, growth medium was switched to low serum differentiation medium consisting of DMEM supplemented with 2% FCS and 2 mg/mL 6‐aminocaproic acid. Differentiation medium was then exchanged every 4 days.

For primary cultures, the same procedure was applied. Growth medium was switched to differentiation medium consisting of Ham's F‐10 supplemented with 20% HS (v/v) and 2 mg/mL 6‐aminocaproic acid. Differentiation medium was then exchanged every 2 days.

### 2.5 Immunofluorescence labelling (Tables S1 and S2)

Microtissues were fixed for 1 h with 3.7% paraformaldehyde at room temperature. Then, microtissues were removed from their support and transferred in a microtube for permeabilisation with 0.5% Triton X‐100/PBS and were gently shaken (300 rpm) at 34°C on an orbital shaker for 2 days. Microtissues were then incubated at 4°C for 1 day in blocking solution PBS, 10% FCS, 1% Triton X‐100, 2.5% dimethyl sulphoxide (DMSO). After washing with PBS, samples were incubated at 15°C on an orbital shaker for 4 days with primary antibody α‐actinin sarcomeric, troponin T or fast myosin heavy chain (MHC) diluted in incubation solution (PBS, 1% FCS, 0.2% Triton X‐100, 2.5% DMSO). After 3 washes for 1 h in PBS, 3% NaCl, 0.2% Triton X‐100 (washing buffer), samples were incubated with FluoProbes® 488 Goat Anti‐Mouse IgG (H + L) antibody in incubation solution for 2 days at 15°C on an orbital shaker. Hoechst at 0.1 mg/mL was used to counterstain cell nuclei. After incubation, samples were washed 4 times for 1 h in washing buffer. The last wash was incubated at 4°C overnight followed by 3 washes for 1 h in PBS the next day; microtissues were then mounted for microscopy imaging.

### 2.6 Tissue clearing and microscopy

Microtissues were mounted with RapiClear® 1.49 reagent in iSpacer microchamber and coverslip. Confocal images were acquired with an inverted Andor Spinning disk CSU‐W1 and collected with a sCMOS Zyla 4.2 camera (Andor) with IQ3 software on MRI‐DBS facility. Immunofluorescence Images were obtained with 20X/0.75 plan‐apo dry objective for Zstack (optimal Zstep of 0.6 μm). Sequential acquisition of light emission was performed with 405 nm and 488 nm lasers and emission was collected with BP emission filters 425–470 nm and 505‐542 nm respectively. Images represents a Z projection of the whole Zstack, processed with open‐source Fiji software (version 1.54p).

#### 2.6.1 Myotube diameters

Width measurements were performed on randomly acquired images of myotubes differentiated for 7, 14, or 21 days. For each myotube, the diameter was determined by calculating the mean of three measurements taken perpendicular to the longitudinal axis of the fused myotube. Measurements were carried out using Fiji software (version 1.54p). A total of 50–100 myotubes were measured from three independent cultures. Histograms represent data expressed as means ± SEM.

### 2.7 Electrical pulse stimulation (EPS)

3D C2C12 myotubes in six‐well dishes were placed in a chamber for electrical stimulation (C‐Dish; IonOptix, Milton, MA). Electrical stimulation was applied to the cells in the C‐Dish using a C‐Pace pulse generator (C‐Pace 100; IonOptix). The cell culture medium was changed to fresh medium before tEPS (1 Hz, 7.8 ms, 40 V).

### 2.8 Gene expression studies (RT‐qPCR)

Total RNA was isolated from two 3D muscle microtissues using TRIzol (15596‐018). RNA concentration was determined by spectrophotometric analysis (BioDrop DUO; BioDrop, Cambridge, UK), and purity was assessed by an OD260nm/OD280nm absorption ratio (>1.8). RNA quality was validated by using 1% agarose gel electrophoresis. Reverse transcription was performed with 2 μg of total RNA and the high‐capacity cDNA Reverse Transcription Kit according to the manufacturer's instructions. One‐tenth of the obtained cDNA was used in each PCR assay. Real‐time quantitative PCR analysis was performed using a Step One Plus detection system (Applied Biosystems, Life technologies, Villebon‐sur‐Yvette, France) with 10 μL of Mastermix SYBR, 10 nmol/L of forward and reverse primers, and 5 μL of diluted cDNA template to a final volume of 15 μL. The forward and reverse primers are listed in Table S3. All PCR assays were performed in duplicate using the following cycling settings: 95°C for 2 min followed by 40 cycles of 95°C for 5 s and 60°C for 30 s. Relative mRNA levels were normalized to the levels of the housekeeping genes *ARP* and *Tuba1α*. The result is expressed using the comparative cycle threshold method to generate ΔΔCt values with template dilutions ranging from 10^1^ to 10^6^ copies. The PCR overall efficiency (E) was calculated from the standard curve slopes according to the equation E = [10^(−1/slope)^] − 1, and this value was >95% for all assays. The relative abundance of each sample was normalized according to the equation: Relative Quantity = 2^−ΔΔCt^. All of the experiments were performed according to the minimum information for publication of quantitative real‐time PCR experiment (MIQE) guidelines (Bustin et al., 2009; Schmittgen & Livak, 2008).

### 2.9 Protein isolation and western blot analysis (Table S2)

3D skeletal muscle microtissues were lysed in RIPA buffer with protease inhibitor cocktail. To facilitate extraction, a pellet micropestle was used. Protein concentrations were determined using the BioRad DC kit. The Western blot protocol was performed as previously described (Rodriguez et al., 2011): Western blots were immunoprobed with following primary antibodies: embryonic myosin heavy chain (MHC), sarcomeric MHC, troponin T and β actin used as loading control. After washing, primary antibodies were detected with peroxidase‐conjugated secondary antibody. Proteins were visualized by enhanced chemiluminescence using a Santa Cruz ECL kit and signals were detected with the ChemiDoc Touch Imaging System (BioRad, 1.1.0.4, 732BR1121). Band intensities were measured with the Image Lab software (BioRad, Version 5.2.1 for Windows 7).

### 2.10 High‐resolution mitochondrial respiration

3D muscles microtissues were rinsed two times in PBS and collected in 1 mL of warm MiR05‐kit respiration medium (0.5 mM ethylene glycol‐bis‐(β‐aminoethyl ether)‐N,N,N′,N′‐tetraacetic acid, 3 mM MgCl_2_, 60 mM Lactobionic acid, 20 mM taurine, 10 mM KH_2_PO4, 20 mM 4‐(2‐hydroxyethyl)‐1‐piperazineethanesulfonic acid [HEPES], 110 mM sucrose, and 1 g/L bovine serum albumin, fatty acid free pH 7.1).

Muscle microtissues were then transferred in two sealed thermostated chambers (37°C) of high‐resolution oxygraph (Oxygraph‐2 k, OROBOROS Instruments, Innsbruck, Austria) containing 0.5 mL (C2C12 cells–sV‐Module) or 2 mL (WT and *Mstn* ^*−/−*^ primary muscle cells) of respiration medium with continuous stirring (750 rpm).

Mitochondrial respiration was assessed with the substrate‐uncoupler‐inhibitor titration (SUIT) protocol SUIT‐008_O2_ce‐pce_D025 (https://wiki.oroboros.at/index.php/SUIT‐008_O2_ce‐pce_D025) (Figure 3a).

Basal respiration (Routine) respiration was measured, followed by addition of digitonin to achieve plasma membrane permeabilisation (15 μg/mL, for primary 3D muscles, none for 3D C2C12 muscles). Mitochondrial pathways respiratory states were then stimulated with the sequential addition of the following substrates and inhibitors: 5 mM pyruvate and 2 mM malate (NADH (N)‐pathway in the LEAK state); 5 mM ADP (N‐pathway in the OXPHOS state); 10 μM cytochrome c (integrity of the outer mitochondrial membrane); 10 mM glutamate (N‐pathway in the OXPHOS state); 10 mM succinate (NADH and Succinate (NS) pathways in the OXPHOS state); titrations (1 μL; 0.5 μM) of chemical uncoupler carbonylcyanide‐chlorophenylhydrazone (CCCP) maximal Electron Transfer System (ETS) capacity (NS‐ETS capacity); 0.5 μM rotenone (inhibition of complex I, S‐ETS capacity); 5 μM antimycin A inhibition of CIII, residual oxygen consumption (ROX) (Gnaiger, 2020) (Figure 3a).

Data acquisition and analysis were performed using Oxygraph‐2 k DatLab software version 7.4.0.4 (OROBOROS Instruments, Innsbruck, Austria). The respiratory control ratio (RCR) was set as the ratio of N‐OXPHOS respiration over N‐LEAK respiration.

### 2.11 Statistical analysis

Data are represented as mean ± SEM from at least three independent experiments. For gene expression analyses, statistical significance was assessed using the non‐parametric Mann–Whitney test to determine the effect of differentiation time. For C2C12 myotube diameters, a non‐parametric Kruskall–Wallis test followed by Dunn's multiple comparisons test was used to determine the effect of differentiation time. For primary myotube diameters, a paired Student's *t*‐test was used to assess the effect of genotype. For mitochondrial respiration, a two‐way ANOVA followed by Sidak's multiple comparisons test was used to evaluate the effects of genotype and respiratory state, while a paired Student's *t*‐test was used to assess the effect of genotype on the respiratory control ratio (RCR). For all analyses, statistical significance was set at *p* < 0.05. Data were analyzed using GraphPad Prism 8 software.

## 3 RESULTS

### 3.1 3D muscle construction: Proof of concept using a murine myoblast cell line (Figure 1a)

**FIGURE 1:**
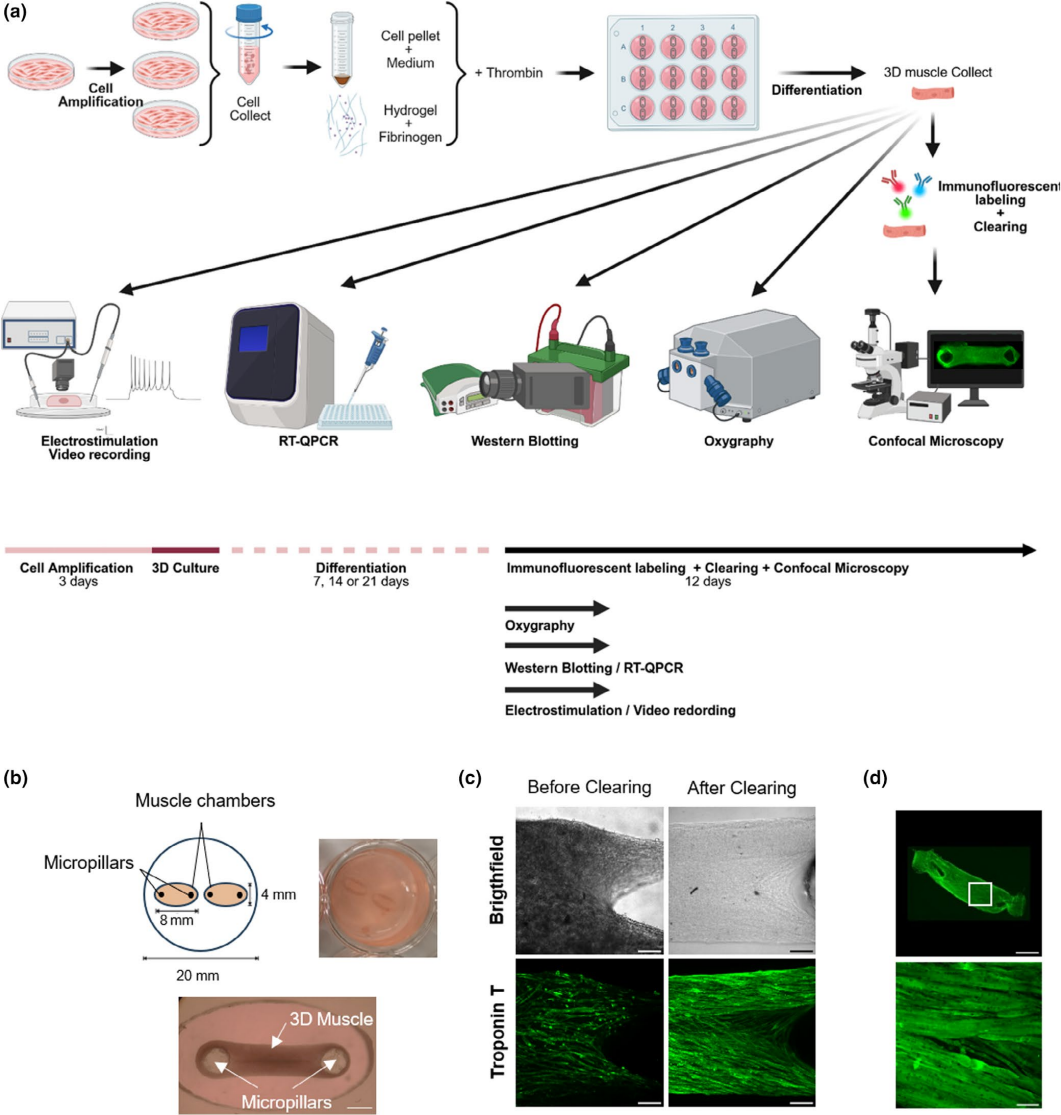
Establishment of 3D C2C12 self‐organized skeletal muscle microtissues. (a) A diagram illustrating the 3D microtissue culture process: Ring‐shaped tissues are constructed and cultured on two micropillars, which serve as anchors during differentiation. The morphological, metabolic, and functional characteristics of the tissues are subsequently analyzed. (b) eNUVIO OMEGA^MP^ devices are assembled in a standard 12‐well plate, allowing for the observation of the resulting 3D skeletal muscle microtissue with a binocular loupe. (c) Brightfield and immunofluorescence images of Troponin T (green) are captured before and after clearing using a spinning‐disk Andor W1 microscope at a magnification of ×10 (scale bar = 200 μm). (d) Confocal immunofluorescence images of C2C12 myotubes on day 14 are stained with anti‐troponin T antibodies (green). Tile scan images at 4× magnification are shown (scale bar = 1000 μm), along with images at 20× magnification (scale bar = 100 μm).

Due to their size and ability to differentiate into myotubes, C2C12 cells (murine myoblasts) were initially used to optimize the cell culture conditions. Figure 1b shows the device selected for building these 3D muscle structures. The Omega^MP^ device (Enuvio) is ideal for creating 3D muscle structures using a hydrogel made from Geltrex and fibrinogen. The success of this culture mainly depends on the quantity of cells and hydrogel nature. We found that a culture of 800,000 cells from the C2C12 line in the proliferation phase was ideal for forming a structure around the two pillars of the device in a few hours. To achieve long‐term stability of these 3D muscle structures, we experimented with different hydrogel compositions and concentrations of Geltrex and fibrinogen.

We were then able to remove our 3D structures from the inserts at various differentiation stages and label them using specific indirect immunofluorescence methods. However, we encountered a thickness problem. Our 3D muscles are around 400 μm thick, which is too thick to stain the full depth of the structure. We therefore used a clearing method to improve both the labelling and image quality. This delipidation method increases the transparency of the 3D microtissue allowing for clearer visualization of the fluorescent staining of the formed myotubes (Figure 1c).

**FIGURE 2:**
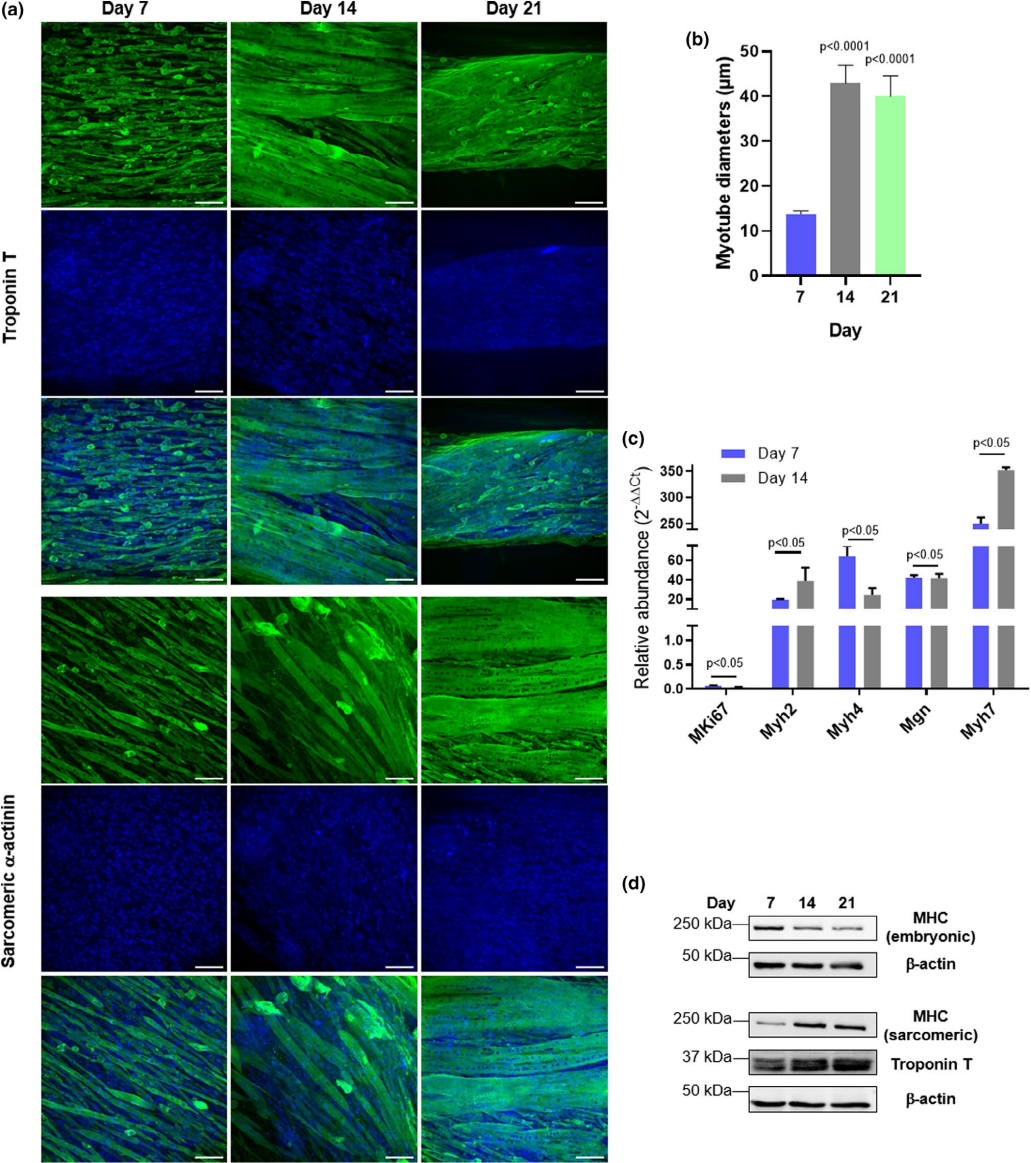
Morphological characterization of 3D C2C12‐type skeletal muscle microtissues on days 7, 14, and 21 (a) Confocal immunofluorescence images of C2C12 myotubes stained with anti‐sarcomeric α‐actinin and anti‐troponin T antibodies (green). Nuclei were visualized by Hoescht staining (blue). Images at 20X magnification (scale bar = 100 μm; independent preparations, *n* = 3). (b) Analysis of the diameters of C2C12 muscle myotubes during differentiation. The histograms represented means ± SEM. At least 50 myotubes at each time of differentiation were analyzed. A Kruskall‐Wallis non‐parametric test followed by Dunns multiple comparison test was used to determine the effect of differentiation time. (c) Analysis of gene expression of 3D myotube markers. Gene expression was calculated as relative abundance (2^−DDCt^) for each condition (*n* = 3). The histograms represented means ± SEM of 3 independent cultures, statistical analysis were performed using a Mann–Whitney non‐parametric test, *p* < 0.05 vs Day 0. Markers: Mki67, Antigen identified by monoclonal antibody Ki‐67; Myh2, myosin heavy chain 2; Myh4, myosin heavy chain 4; Mgn, myogenin; Myh7, myosin heavy chain 7. (d) Western blot analysis showing myosin heavy chain (MHC) isoforms (embryonic and sarcomeric), troponin T and β‐actin (as a loading control). Each western blot lane contained 50 μg of protein extracted from two pools of 3 independent 3D microtissues (6 total microtissues).

The C2C12 myogenic differentiation in 3D culture was evaluated by analyzing the expression of muscle‐specific markers using immunocytochemistry, RT‐qPCR and Western blotting analyses (Figure 2). We monitored the development of the 3D structures by immunofluorescence analysis using anti‐troponin T and anti‐sarcomeric α‐actinin antibodies to assess sarcomeric organization at 7, 14, and 21 days of differentiation (Figure 2a). Within 3 weeks of differentiation, multi‐nucleated and branched myotubes were observed. At day 7, only a few thin but well‐organized myotubes were detected. By day 14, myotube size had markedly increased. This maturation process continued through day 21. Myotube diameters were quantified at days 7, 14 and 21 of differentiation (Figure 2b), confirming as a progressive increase in myotube size over time. Notably, myotubes can be seen throughout the 3D structure, which was approximately 400 μm thick (Data S4). At the transcriptional level, the expression of genes encoding markers of proliferation and differentiation was analyzed in 3D myotubes at days 0, 7, and 14 (Figure 2c). The 3D structures showed over‐expressed myosin heavy polypeptides 2, 4 and 7 (Myh2, Myh4 and Myh7), as well as myogenin (Mgn) together with reduced gene expression of the proliferation marker Mki67. The expression of troponin T and the sarcomeric myosin heavy chain, as well as the evolution of the embryonic myosin heavy chain were then investigated using western blotting (Figure 2d). Over time in 3D culture, muscle tissues upregulate the expression of the sarcomeric myosin heavy chain (MHC), accompanied by downregulation of embryonic MHC expression. Collectively, these data suggest that 3D culture induces myogenic differentiation accompanied with gradual sarcomere structural maturation.

**FIGURE 3:**
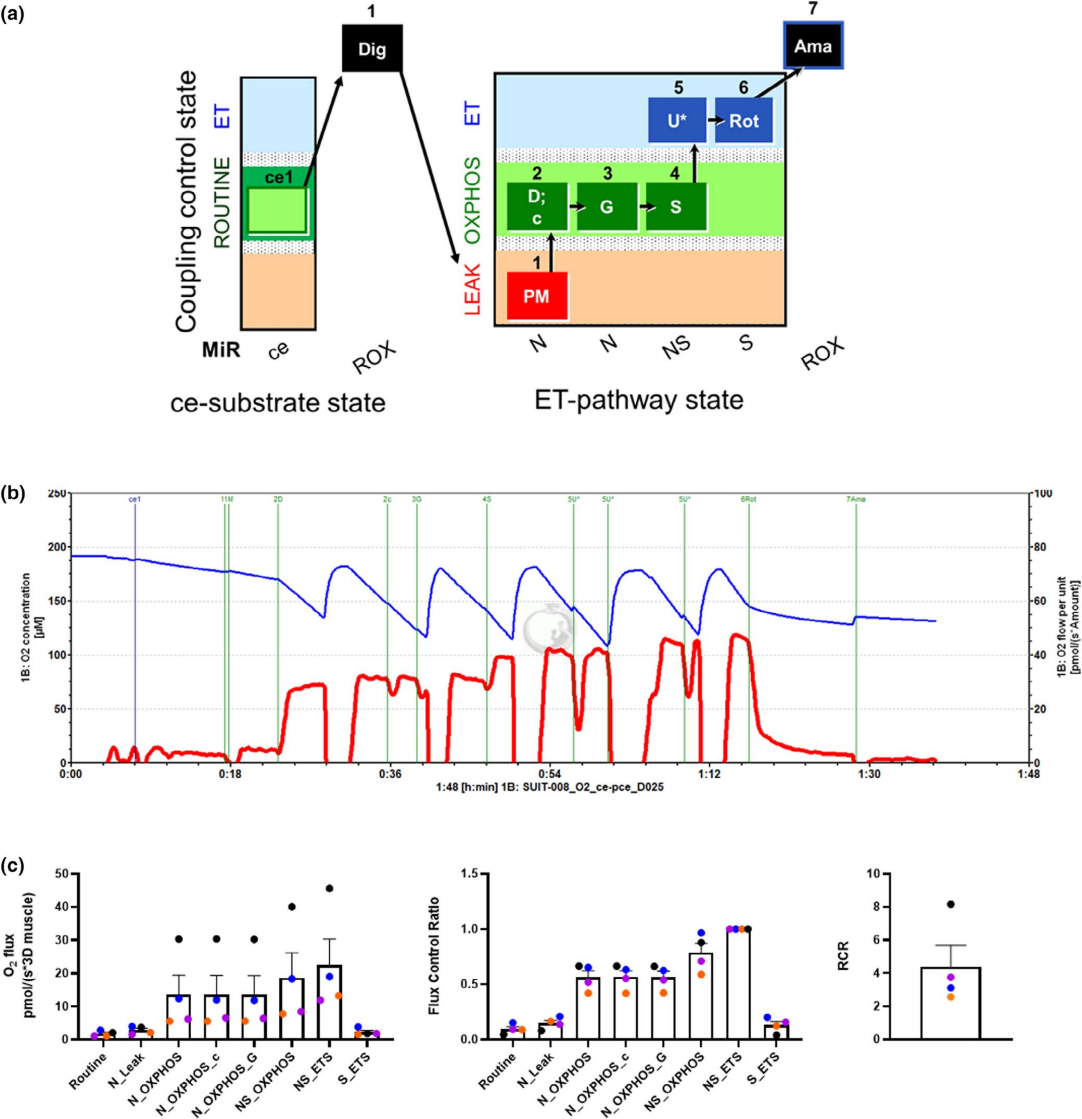
Mitochondrial respiration of 3D C2C12‐type skeletal muscle microtissues on day 14. (a) Substrate‐uncoupler‐inhibitor titration protocol (SUIT‐008_O2_ce‐pce_D025). Sequential titrations and respiratory states. 1ce1: Routine (basal) cell respiration R in living cells. 1Dig: Plasma membrane permeabilisation with digitonin. 1 pm: NADH‐pathway (N‐pathway) in the presence of 5 mM pyruvate and 2 mM malate in the N‐LEAK state. 2D: Addition of saturating ADP (5 mM) (N‐OXPHOS state). 2c: 10 μM cytochrome c for evaluating the integrity of the outer mitochondrial membrane (N‐OXPHOS‐c state). 3G: 10 mM glutamate as an additional NADH‐linked substrate (N‐OXPHOS‐G state). 4S: NS‐pathway evaluation with 10 mM succinate, (NS‐OXPHOS state). 5 U: Uncoupler titrations to evaluate the electron transfer system (ETS) capacity (NS‐ETS state). 6Rot: Inhibition of CI by 0.5 μM rotenone and evaluation of succinate pathway (S‐ETS state). 7Ama: Inhibition of CIII by 5 μM antimycin A (residual oxygen consumption, ROX). (b) Representative respiration trace with 3D C2C12 muscle following SUIT‐008_O2_ce‐pce_D025. (c) Mitochondrial respiration is expressed as oxygen flux per microtissue [pmol·s^−1^·amount] and as flux control ratios (FCR) of respiratory states over electron transfer system (ETS) capacity. The respiratory control ratio (RCR) was set as the ratio of N‐OXPHOS respiration over N‐LEAK respiration. Results represented mean ± SEM of 4 independent cultures.

To assess the functionality of the 3D microtissue, high‐resolution respirometry was used to measure mitochondrial activity as a proxy for metabolic activity in the 3D microtissue (Figure 3a). As expected, sequential addition of substrates from the NS‐pathway progressively increased mitochondrial respiration (Figure 3b), and maximal respiration stimulated by the addition of CCCP ranged from ~13 to 45 pmol·s^−1^ per microtissue. The flux control ratio showed that the mitochondrial respiratory pathways were stimulated to the same extent in all four independent experiments involving microtissue preparation (Figure 3c). The respiratory control ratio (RCR), which evaluates the coupling between respiration and ATP production, ranged from ~2.5 to 8.

Finally, another way to check functionality was to stimulate the 3D structures electrically. Stimulating the 3D muscles at 1 Hz for 7.8 ms at 40 V caused visible muscle contractions. Videos of contractile activity are publicly available on the Recherche Data Gouv repository under the following filenames: S5A_Supplemental_Data_Video_Records_of_Microtissue_Contractility.mp4 (C2C12 cells) and S5B (*Mstn* ^*−/−*^ model), at https://doi.org/10.57745/VDA5YN.

### 3.2 Skeletal muscle cells retain the *Mstn* ^*−/−*^ phenotypic and metabolic features in 3D culture

Following optimisation of the 3D culture conditions using the C2C12 cell line, the technology was extended to a 3D myotube model based on primary cultures derived from *Mstn* ^*−/−*^ mice, in comparison with wild‐type (WT) mice. *Mstn* ^*−/−*^ mice exhibit a hyper‐muscular phenotype compared to WT mice. The same conditions as for the C2C12 cells were applied: 800,000 satellite cells were required to form a 3D structure with the *Mstn* ^*−/−*^ muscle primary culture.

**FIGURE 4:**
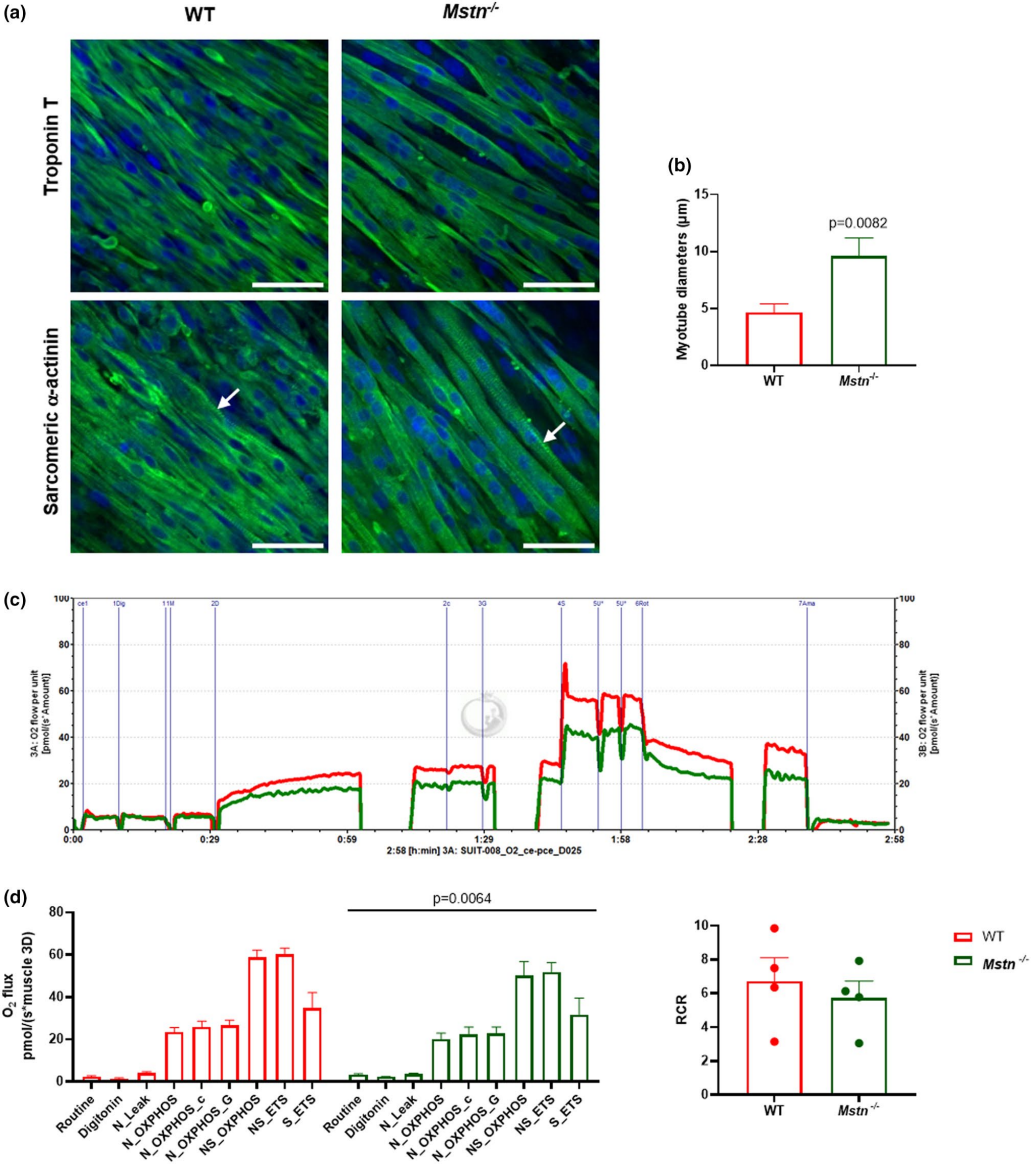
Morphological characterization and mitochondrial respiration of 3D skeletal muscle microtissues derived from primary mouse myotubes on day 14. (a) Confocal immunofluorescence images of 3D primary muscle myotubes, stained with anti‐troponin T and anti‐sarcomeric α‐actinin antibodies, at 20× magnification (scale bar = 50 μm for the zoomed images; *n* = 3 independent culture). Nuclei were revealed by Hoescht staining (blue). The arrow indicates the organized sarcomere structures in this myotube. (b) Analysis of myotube diameters of WT and *Mstn* ^−/−^ 3D primary muscle. Histograms are means ± SEM for three independent cultures. At least 100 myotubes stained with anti‐sarcomeric α‐actinin antibody were analyzed for WT and primary muscles. A paired Student *t*‐test was used to determine genotype effect. (c) Representative respiration trace with WT (red) and *Mstn* ^−/−^ (green) 3D primary muscle following SUIT‐008_O2_ce‐pce_D025. (d) Analysis of mitochondrial respiration of WT (red) and *Mstn* ^−/−^ (green) 3D primary muscles. Mitochondrial respiration is expressed as oxygen flux per microtissue [pmol·s^−1^·amount]. The respiratory control ratio (RCR) was set as the ratio of N‐OXPHOS respiration over N‐LEAK respiration. Results represented mean ± SEM of 4 independent cultures, statistical analysis for genotype effect was performed using a two‐way ANOVA. See Figure 3a and Methods section for respiratory states legend.

Myotube‐specific proteins (α‐actinin sarcomeric and troponin T) were labeled after 14 days of 3D culture (Figure 4a). At higher magnification, the sarcomeres can be distinguished, indicating that the myotubes have undergone advanced differentiation. Structures made from the primary culture of *Mstn* ^*−/−*^ muscles contain larger myotubes than those formed from WT cultures. Indeed, myotube diameters were statistically twofold greater in *Mstn* ^*−/−*^ 3D cultures compared with WT 3D cultures (Figure 4b).

Regarding mitochondrial functionality, NS‐pathway respiratory states were generally lower in microtissues from the *Mstn* ^*−/−*^ primary culture compared than in WT culture (*p* < 0.01), with a decrease of up to 15% observed in coupled NS‐OXPHOS and uncoupled NS‐ETS respiratory states. Although RCR was higher in the primary culture than in C2C12 cells, it did not differ between WT and *Mstn* ^*−/−*^ muscle microtissues (Figure 4c,d).

Similar to the 3D structures obtained from the C2C12 line, those derived from the primary *Mstn* ^*−/−*^ myoblast cultures also exhibited contractile activity (Data S5).

## 4 DISCUSSION

A major challenge in creating three‐dimensional (3D) microtissue skeletal muscle models is approximating the complexity of native muscle physiology. This study provides original evidence that 3D models based on either an immortalized murine myogenic cell line or satellite cells can generate skeletal muscle tissues that display measurable mitochondrial function, as well as structural and contractile properties. Notably, 3D myotubes derived from *Mstn* ^*−/−*^ mice exhibit key features of the in vivo phenotype, such as muscle hypertrophy accompanied by reduced mitochondrial respiration (Gu et al., 2022; Ploquin et al., 2012).

### 4.1 Generation of a 3D model using cell lines or primary satellite cells allows the measurement of metabolic characteristics in a controlled environment, such as mitochondrial respiration

In this study, we developed and validated a 3D skeletal muscle microtissue using the C2C12 murine myoblast cell line. We then adapted this model to primary cultures derived from *Mstn* ^*−/−*^ mice. Optimizing the cell‐to‐hydrogel ratio (Geltrex–fibrinogen) enabled stable microtissues to form rapidly at the Omega^MP^ device pillars. Immunofluorescence (sarcomeric α‐actinin and troponin T), Western blot (upregulation of sarcomeric myosin heavy chain and downregulation of embryonic myosin heavy chain) and gene expression analyses (upregulation of myosins) confirmed progressive myotube maturation over the course of the 21‐day culture period. These findings are consistent with previous reports demonstrating that 3D organization promotes myotubes alignment, fusion, and contractile functionality (Afshar et al., 2020; Afshar Bakooshli et al., 2019; Wells‐Cembrano et al., 2022).

Notably, the ability to perform high‐resolution respirometry on intact 3D skeletal muscle microtissues represents a methodological advancement. Previous in vitro studies have mostly relied on isolated mitochondria or trypsinised myotubes (Robinson et al., 2019; Younis et al., 2023) which disrupt cellular architecture and may alter mitochondrial behaviour. In contrast, our model allows us to assess mitochondrial respiration within a structured and contractile tissue while preserving cell–cell interactions and cytoskeletal organization. These factors are known to influence mitochondrial dynamics and function. It should be noted that mitochondrial respiratory function assessed by RCR calculation was found in 3D C2C12 microtissues, in the same range as those previously reported in 2D C2C12 myotubes (Kumar et al., 2021; Kumar et al., 2024).

Mitochondrial function plays a key role in ATP production, muscle contraction and the regulation of hypertrophy and atrophy. As mitochondrial dysfunction is a hallmark of numerous pathological and aging‐related conditions, there is a clear need for physiologically relevant models to investigate muscle metabolism and identify bioactive compounds capable of restoring mitochondrial activity. Unlike conventional two‐dimensional (2D) cultures, 3D engineered muscle constructs more accurately replicate the structural organization, maturation state and mechanical environment of native muscle, all of which are critical influences on mitochondrial activity. In this context, our 3D muscle model provides a controlled experimental system in which mitochondrial respiration can be reliably assessed in a tissue‐like environment.

### 4.2 3D culture in primary cells culture maintains the phenotypic characteristics of the animal

The absence of myostatin is associated with pronounced muscle hypertrophy and impaired mitochondrial function (Amthor et al., 2007). Previous work from our laboratory has shown that *Mstn* ^*−/−*^ mice exhibit significant functional defects in intermyofibrillar mitochondria. This subpopulation of mitochondria is a mitochondrial subpopulation involved in sustaining muscle contraction (Ploquin et al., 2012). These defects include reduced mitochondrial respiratory coupling efficiency, resulting in increased oxygen consumption for a given amount of ATP produced. In this study, we investigated whether mitochondrial respiratory alterations associated with myostatin deficiency persist in a 3D culture context. This hypothesis was experimentally validated using high‐resolution respirometry in 3D *Mstn* ^*−/−*^ myotubes.

Primary cultures derived from *Mstn* ^*−/−*^ mice exhibited key in vivo phenotypic characteristics, such as enlarged myotubes, as demonstrated by sarcomeric α‐actinin staining. Furthermore, there was a significant decrease in coupled and uncoupled mitochondrial respiration compared to wild‐type controls, while the respiratory control ratio remained unchanged. Taken together, these results demonstrate that hypertrophy and associated metabolic alterations induced by myostatin deficiency are preserved in the 3D culture system. A reduction in mitochondrial content has been reported in *Mstn* ^*−/−*^ muscle (Amthor et al., 2007; Lipina et al., 2010; Savage & McPherron, 2010), which may account for the mitochondrial respiratory profile observed in 3D *Mstn* ^*−/−*^ myotubes.

To the best of our knowledge, this is the first study to report measurements of mitochondrial respiration using a functional bioengineered 3D skeletal muscle model. We showed decreased mitochondrial respiration in *Mstn* ^*−/−*^ 3D myotubes, despite increased activity having previously been reported in intermyofibrillar mitochondria isolated from *Mstn* ^*−/−*^ muscle *in vivo*. These discrepancies emphasize the significant impact of the experimental context on mitochondrial phenotyping. *in vivo* muscle contains distinct mitochondrial subpopulations that are regulated differently by factors such as mechanical load, innervation, and systemic factors like hormones and substrate availability (Chabi et al., 2008; Picard et al., 2015). In contrast, 3D *in vitro* systems isolate muscle‐intrinsic properties while excluding neural and endocrine regulation, which may partly explain the observed differences in mitochondrial activity.

Beyond mechanistic investigations, 3D muscle models provide valuable platforms for screening drugs and molecules. They can be created using patient‐derived muscle biopsies, enabling the study of muscular pathologies in a controlled environment (Berry et al., 2023). Mitochondrial respiration analyses have already been successfully performed in 3D cardiac muscle models (Pocock et al., 2024).

More broadly, 3D muscle culture systems are a significant improvement on traditional 2D cell cultures because they more accurately replicate the structure and mechanical properties of native skeletal muscle. These models provide a physiologically relevant framework for exploring the cellular and molecular mechanisms involved in muscle development, regeneration, and disease progression (Rajabian et al., 2021), and are particularly valuable for pharmacological applications (Langhans, 2018). Furthermore, 3D muscle constructs can be generated from patient‐derived biopsies or induced pluripotent stem cells, enabling the development of personalized disease models. Such an approach has successfully reproduced disease‐specific phenotypes; for example,r in the case of facioscapulohumeral muscular dystrophy, where iPSC‐derived 3D cultures have been found to be more functionally relevant than conventional 2D systems (Franken et al., 2025).

### 4.3 Perspectives and limitations

Although several studies have described 3D culture systems, many of these models rely on complex, custom‐made, and poorly standardized devices, which may limit reproducibility and broader implementation. In the present study, we used eNUVIO OMEGA^MP^ inserts, a user‐friendly and standardized device that enables the generation of aligned, contractile 3D muscle microtissues resembling native skeletal muscle. Using this system, C2C12 myoblasts or mouse muscle‐derived satellite cells were induced to undergo skeletal myogenesis within a biocompatible hydrogel according to established protocols. Importantly, the main novelty of our work lies in the development of methods to assess mitochondrial respiration directly in these engineered muscle constructs. Furthermore, we demonstrate that this 3D device faithfully reproduces the metabolic characteristics associated with the myostatin phenotype. This added value underscores its potential as a relevant model for investigating skeletal muscle metabolism and for identifying therapeutic compounds targeting muscle metabolic pathways.

Despite these promising results, several limitations should be acknowledged. For example, tissue thickness (approximately 400 μm) may restrict the diffusion of oxygen and nutrients, while the absence of supporting cell types such as fibroblasts or endothelial cells may hinder physiological maturation. Furthermore, prolonged mechanical or electrical stimulation could further improve sarcomeric organization and functional performance. The incorporation of co‐culture strategies and mechanical stimulation, as recently demonstrated (Iberite et al., 2023), could enhance tissue maturation and contractile physiology even further.

Finally, 3D muscle tissues offer an ethical and physiologically relevant alternative to animal models, aligning with the “3Rs” principle (Replacement, Reduction, Refinement) promoted by the OECD and the European Union. The European Medicines Agency (EMA) actively encourages the development and use of human‐relevant in vitro models to improve the prediction of drug efficacy and toxicity (Westmoreland et al., 2022). Future developments of this model could include integrating vascular‐like networks or motor neuron co‐cultures to better reproduce oxygen delivery and neuromuscular coupling. Longitudinal studies combining mechanical loading with metabolic stress could provide further insight into how mitochondrial plasticity adapts to functional demand in engineered muscle tissues.

## 5 CONCLUSION

In summary, this study shows that bioengineered 3D skeletal muscle tissues are a reliable and physiologically relevant model for studying muscle metabolism. By facilitating the evaluation of mitochondrial respiration within a structured and contractile tissue, this model paves the way for new approaches to mechanistic studies and therapeutic screening in the field of skeletal muscle biology.

## AUTHOR CONTRIBUTIONS

**Barbara Vernus:** Conceptualization; data curation; formal analysis; investigation; methodology; resources; validation. **Elodie Jublanc:** Data curation; formal analysis; methodology; validation. **Béatrice Chabi:** Data curation; formal analysis; investigation; methodology; validation. **Laurence Pessemesse:** Investigation; methodology. **Zacharie Cheng‐Boivin:** Conceptualization; methodology. **Benoit J. Gentil:** Conceptualization; methodology. **Anne Bonnieu:** Conceptualization; formal analysis; methodology; validation. **Christelle Koechlin‐Ramonatxo:** Conceptualization; funding acquisition; validation. **Bénédicte Goustard:** Conceptualization; data curation; formal analysis; funding acquisition; investigation; methodology; supervision; validation; visualization.

## FUNDING INFORMATION

We thank an AlimH department grant of INRAE (ANSSD‐2022) for funding these studies. We thank the CNES (Centre National d'Etudes Spatiales) (8502‐4800001211) for funding these studies and, more particularly, Guillemette Gauquelin‐Koch for providing significant support for this research project. Zacharie Cheng‐Boivin is supported by a Fonds de Recherche du Québec en Santé.

## CONFLICT OF INTEREST STATEMENT

The authors declare that they have no conflicts of interest.

## ETHICS STATEMENT

In accordance with institutional and national guidelines, no ethical approval was required, as no experimental procedures were carried out on animals. The mice came from our own breeding programme and were not subjected to any experimental procedures.

## Supporting information


**Table S1:** Immunofluorescence solution.
**Table S2:** List of antibodies used for immunofluorescence assays (IF) and western blotting (WB).
**Table S3:** Primers for gene expression in 3D muscle.
**Data S4:** Zstack video.
**Data S5:** Video records of microtissues contractility.

## Data Availability

The data that support the findings of this study are openly available in Recherche Data Gouv at https://doi.org/10.57745/VDA5YN.
