# Supplementary material for: 3D skeletal muscle model recapitulating the myostatin knockout phenotypic and mitochondrial metabolic features
Source: Physiol Rep. 2026 Jun 16;14(12):e70947. doi: 10.14814/phy2.70947 (PMC13273021; doi:10.14814/phy2.70947)
Supplement: Supplementary file 1 — Table S1: Immunofluorescence solution. Table S2: List of antibodies used for immunofluorescence assays (IF) and western blotting (WB). Table S3: Primers for gene expression in 3D muscle. Data S4: Zstack video. Data S5: Video records of microtissues contractility. [file PHY2-14-e70947-s001.zip › S1-S3 Supplemental Tables.pdf]

S1 supplemental Table: Immunofluorescence solutions

Name	Details
Blocking solution	PBS, 10% FCS, 1% Triton X-100, 2.5% Dimethyl sulphoxide (DMSO)
Incubation solution	PBS, 1% FCS, 0.2% Triton X-100, 2.5% DMSO
Washing buffer	PBS, 3% NaCl, 0.2% Triton X-100

S2 supplemental Table: List of antibodies used for immunofluorescence assays (IF) and western blotting (WB)

Antibody	Supplier	Reference	Dilution	Application
β-actin	Santa Cruz	sc-81178	1/200	WB
α-actinin sarcomeric	Sigma	A7811	1/200	IF
Myosin heavy chain (MHC), embryonic	DSHB	F1.652	1/50	WB
Myosin heavy chain (MHC), sarcomere	DSHB	MF20	1/50	WB
Troponin T	Sigma	T6277	1/2000	WB
FluoProbes® 488 Goat Anti-Mouse IgG (H+L)	Interchim	FP-SA4000	1/50	IF
Anti-mouse IgG, HRP-linked	Cell Signaling	7076	1/2500	WB

S3 supplemental table. Primers for gene expression in 3D muscle : sequences of primers (5'-3')

Gene	RefSeq/Gene ID	Forward	Reverse
Myh7	NM_080728	ATAGCAGGAAAGGGGCTGA	GCCTCTCATCACGCATCTC
Myh2	NM_001039545	AGGCGGAAAGAAGCTACCAT	GTGGTGATCAGCAGCATTTC
Myh4	NM_010855.3	CTGCAGGACTTGGTGGACA	CTTGGCCAGGTTGACATTG
Mgn	NM_002479.6	CCCTGAAGAGAAGCACCCTG	ATGTACTGGATGGCACTGCG
Pcna	NM_011045.2	AGGCACTCAAGGACCTCATC	GAGTCCATGCTCTGCAGGTT
Mki67	NM_001081117.2	TGCAGCAGATGGAACTAGGC	CACTGTGATCTTCCAGCGGT
α-tub	NM_011653	CTGGAACCCACGGTCATC	GTGGCCACGAGCATAGTTATT
Arp	NM_007475.5	ACTGGTCTAGGACCCGAGAAG	TCCCACCTTGTCTCCAGTCT

Myh7, myosin heavy polypeptide 7; Myh2, myosin heavy polypeptide 2; Myh4, myosin heavy polypeptide 4; Mgn, Myogenin; Pcna, proliferating cell nuclear antigen; Mkli67, antigen identified by monoclonal antibody Ki 67; α-tub, alpha-tubulin; Arp, Acidic ribosomal phosphoprotein P0.
